# Prospective multicenter study of murmur localization and stage-associated murmur grading in dogs with echocardiographically diagnosed cardiac diseases: the LISTEN study

**DOI:** 10.3389/fvets.2026.1944439

**Published:** 2026-10-13

**Authors:** Gerhard Wess, Jens Häggström, Julia Stadler, Lara König, Katarzyna Kraszewska, Doris Bittenecker, Oriol Domenech, German Santamarina, Gregory Van den Noortgate, Mikkel Brandt Bjerknaes, Jenny Eberhard, Karin Kriström, Liva Vatne, Michał Gajewski, Christel Schinner-Peters, Guy Tater, Marin Torti, Christoph Schummer, Ingrid Ljungvall

**Affiliations:** 1 LMU Small Animal Clinic, Ludwig-Maximilians-University, Munich, Germany; 2 Swedish University of Agricultural Sciences, Uppsala, Sweden; 3 Vetcardia Veterinary Clinic, Warsaw, Poland; 4 Diagnostic Center for Small Animals, Vienna, Austria; 5 Anicura Istituto Veterinario Novara, Novara, Italy; 6 Institute for Research in Global Health and Sustainable Development (iTERRA), Campus Terra, Universidad de Santiago de Compostela, Lugo, Spain; 7 Dierenartsenpraktijk Akuut, Kortenberg, Belgium; 8 Tierklinik Elversberg, Spiesen-Elversberg, Germany; 9 Evidensia Södra Djursjukhuset Kungens Kurva, Stockholm, Sweden; 10 Norwegian University of Life Sciences, and Empet Nydalen, Oslo, Norway; 11 Mobile Konsultationspraxis für Kleintierkardiologie, Kemnath, Germany; 12 Tierklinik Stommeln, Pulheim, Germany; 13 Clinic for Internal Medicine, Faculty of Veterinary Medicine, University of Zagreb, Croatia; 14 Boehringer-Ingelheim, Ingelheim, Germany

**Keywords:** canine cardiology, cardiac auscultation, disease staging, echocardiography, heart sounds

## Abstract

**Background:**

Cardiac auscultation remains fundamental in dogs with suspected cardiac disease, but large prospective datasets describing clinician-recorded murmur localization and intensity across echocardiographic diagnoses are limited. This study aimed to characterize point of maximal intensity (PMI) patterns in dogs with echocardiographically diagnosed cardiac abnormalities and to evaluate the association between murmur grade and disease stage in dogs with myxomatous mitral valve disease (MMVD) and dilated cardiomyopathy (DCM).

**Methods:**

This prospective international multicenter observational study included 6,996 client-owned dogs examined by 50 cardiology-trained veterinarians. Dogs underwent standardized cardiac auscultation at four predefined thoracic locations and transthoracic echocardiography during the same clinical visit. PMI localization was described across cardiac diagnoses, and murmur grade was evaluated in relation to disease stage in MMVD and DCM.

**Results:**

Dogs with MMVD and DCM predominantly had a left apical PMI, whereas congenital outflow tract diseases and patent ductus arteriosus were predominantly associated with a left basilar PMI. Tricuspid regurgitation and ventricular septal defect were more commonly associated with right apical and right basilar PMIs, respectively. In MMVD, murmur intensity increased with advancing disease stage (*p* < 0.001), and dogs with a murmur greater than soft had higher odds of being in stage B2 or C/D than B1 (OR 35.9, 95% CI 27.6–46.6). In DCM, 25.8% of dogs had no audible murmur. Although murmur grade distribution differed across DCM stages (*p* = 0.03), intensity did not show a consistent stage-associated increase.

**Conclusion:**

Clinician-recorded four-point auscultation identified characteristic regional PMI patterns across several canine cardiac diseases. Murmur grade was strongly associated with disease stage in MMVD but provided limited stage-related information in DCM. Auscultation may support initial clinical assessment and prioritization of differential diagnoses, but echocardiography remains essential for definitive diagnosis and staging.

## 1 Introduction

Cardiac auscultation remains a fundamental component of the cardiovascular examination in dogs and is typically the first diagnostic step in the evaluation of suspected cardiac disease. Established auscultation principles describe characteristic point of maximal intensity (PMI) patterns, with murmurs associated with mitral regurgitation predominantly localized to the left heart apex and murmurs associated with congenital outflow tract abnormalities most commonly heard at the left heart base (1–13). Right-sided murmurs are typically associated with conditions such as ventricular septal defects (14) and tricuspid regurgitation (15). These principles guide clinical decision-making and inform the selection of subsequent diagnostic tests.

Despite its central role, cardiac auscultation has well-recognized limitations. Heart murmur intensity does not consistently reflect disease severity, and certain cardiac diseases, most notably dilated cardiomyopathy (DCM), may be associated with absent or subtle murmurs even in advanced stages (10, 13, 16). Conversely, dogs with early-stage myxomatous mitral valve disease (MMVD) may have echocardiographically detectable mitral regurgitation (MR) without an audible murmur (17–19). Thus, auscultatory findings differ substantially between valvular and myocardial disease and should not be interpreted as a uniform measure of disease presence or severity.

Recent technological developments have facilitated standardized auscultation and the collection of multicenter datasets, enabling more systematic evaluation of auscultatory findings across diverse patient populations (20–24). Although the expected localization of many canine murmurs is well established, large prospective studies that quantify how consistently these patterns occur across a broad range of echocardiographic diagnoses and centers remain limited. Likewise, the association between murmur grade and clinically assigned disease stage has not been comprehensively evaluated in large multicenter cohorts spanning distinct pathophysiologic categories such as valvular and myocardial disease.

The objectives of this prospective multicenter study were therefore (1) to systematically characterize disease-associated PMI localization patterns during cardiac auscultation at four predefined thoracic locations in dogs with echocardiographically diagnosed cardiac abnormalities and (2) to evaluate the associations between murmur grade and disease stage in dogs with MMVD and DCM. We hypothesized that PMI patterns would be disease-associated and that murmur intensity would increase with advancing MMVD stage but show limited association with stage in dogs with DCM.

## 2 Materials and methods

### 2.1 Study design

This was a prospective, international multicenter observational study. Client-owned dogs undergoing clinical cardiac evaluation were prospectively enrolled. The study involved only procedures performed as part of routine clinical care, and no owner-identifiable information was collected in agreement with Regulation (EU) 2016/679 (General Data Protection Regulation). All procedures were performed as part of routine clinical care. Examinations were performed by experienced veterinarians with advanced training in cardiology, including board-certified specialists (ACVIM or ECVIM), residents in training, and clinicians with nationally recognized cardiology qualifications.

Centers were enrolled in two phases: an *initial phase* followed by an *expansion phase* including additional participating centers. Cardiac auscultation and echocardiographic examinations were performed during the same clinical visit.

### 2.2 Dogs

Client-owned dogs presented for cardiac evaluation as part of routine clinical practice were eligible for inclusion at participating centers across Germany, Sweden, Poland, Belgium, Austria, Italy, Spain, the United States, Norway, France, Denmark, Bulgaria, Croatia, and Greece.

Dogs were included if a complete echocardiographic examination and standardized cardiac auscultation had been performed. Dogs without an audible murmur that underwent echocardiography for various clinical reasons, including breed or pre-breeding cardiac screening, pre-anesthetic cardiac assessment, evaluation of clinical signs potentially referable to cardiovascular disease, arrhythmias, abnormal previous diagnostic findings, or follow-up of known or suspected cardiac disease, were also eligible for inclusion. No restrictions were applied with respect to signalment or disease status. If dogs had been examined multiple times, only the results from the first examination were included in the final analysis.

Breed information was not consistently available in the *initial phase* of the study and was therefore prospectively collected only for dogs enrolled during the *expansion phase*. Consequently, breed distribution analyses were restricted to this subset and include fewer cases than the overall study population. Breed distributions were analyzed descriptively and were not intended to estimate breed-specific disease prevalence.

### 2.3 Cardiac auscultation

#### 2.3.1 Stethoscope systems and investigator training

Before participation, all recruiting veterinarians received written study instructions and attended an online training session in which the study design, standardized auscultation protocol, four predefined auscultation sites, murmur grading, PMI recording, and data entry procedures were explained. No formal interobserver calibration test was performed. The study was conducted in two consecutive phases. In the initial phase, cardiac auscultation was performed using a 3M™ Littmann^®^ Cardiology IV stethoscope (3M Company, St. Paul, MN, USA) equipped with an Ekuore digital attachment (Chip Ideas Electronics S.L., Burjassot, Spain). In the subsequent expansion phase, additional centers were enrolled and auscultation was performed using a 3M™ Littmann^®^ CORE Digital Stethoscope (3M Company & Eko Health, St. Paul, MN & Emeryville, CA., USA). For the present analysis, data from both phases were combined into a unified dataset. Both systems allowed digital recording of heart sounds. To ensure consistency across study phases, murmur detection, PMI localization, and murmur grading were performed uniformly using conventional (non-amplified) auscultation.

#### 2.3.2 Auscultation sites and PMI recording

Cardiac auscultation was systematically performed in unsedated dogs at four predefined thoracic locations: left heart apex (LHA), left heart base (LHB), right heart apex (RHA), and right heart base (RHB). These regions were defined anatomically as the left caudoventral precordium over the mitral valve area/apex beat (LHA), the left craniodorsal precordium over the pulmonic and aortic valve areas (LHB), the right caudoventral precordium over the right apical region (RHA), and the right craniodorsal precordium over the right basilar region (RHB). These landmarks correspond to conventional canine cardiac auscultation areas and were adjusted for body size and thoracic conformation. Counting of individual intercostal spaces was not required by the study protocol.

When an audible heart murmur was present, the PMI was recorded as the predefined thoracic region in which the murmur was perceived to be loudest by the attending clinician. For analyses of PMI localization, only dogs with an audible heart murmur (grade > 0) were included. In dogs without an audible heart murmur (grade = 0), no PMI was defined.

#### 2.3.3 Heart murmur grading

To ensure consistency across centers and devices, murmur grading was performed without digital amplification. Heart murmurs were graded by the attending clinician using the conventional six-grade Levine scale (16). Grade 0 was assigned when no murmur was audible. Grade 1 described a very soft murmur audible only after careful auscultation, and grade 2 a soft but readily audible murmur. Grade 3 described a moderately loud murmur without a palpable precordial thrill. Grade 4 described a loud murmur, whereas grades 5 and 6 described very loud murmurs with a palpable thrill, with grade 6 audible with the stethoscope slightly lifted from the thoracic wall. For analysis, grades 1–2 were grouped as soft murmurs, grade 3 as moderate murmurs, and grades 4–6 as loud murmurs, based on a simplified categorization adapted from Ljungvall et al. (7).

### 2.4 Echocardiographic examination and diagnosis

All dogs underwent transthoracic echocardiography, including two-dimensional imaging, color Doppler, and spectral Doppler assessment, using standard right and left parasternal views; subcostal views were obtained when clinically indicated. The specific indication for echocardiography was not recorded as a standardized study variable.

Sedation was permitted for echocardiography if necessary, but sedation status and drug protocols were not recorded as standardized study variables. Cardiac auscultation was performed before echocardiography and before any sedation potentially used for echocardiographic examination. Cardiac diagnoses were established by the attending clinician according to current disease-specific consensus statements and echocardiographic recommendations (12, 17, 18). Depending on the underlying disease, diagnosis was based on the combination of two-dimensional morphology, chamber dimensions, Doppler findings, and functional indices interpreted in the context of the complete echocardiographic examination. For DCM, diagnosis was based on the presence of left ventricular systolic dysfunction together with left ventricular enlargement using normalized M-mode and/or Simpson's method of disks (SMOD) measurements interpreted according to current recommendations, after exclusion of other conditions capable of producing a DCM phenotype (12, 19).

### 2.5 Classification based on echocardiographic findings

Dogs were classified based on echocardiographic findings as having either cardiac abnormalities or no structural cardiac disease. Cardiac abnormalities comprised structural cardiac diseases as well as non-structural cardiac conditions associated with abnormal echocardiographic findings. Structural cardiac diseases included MMVD, DCM and congenital heart disease, including pulmonic stenosis, patent ductus arteriosus, subaortic/aortic stenosis, ventricular septal defect, mitral valve dysplasia, and tricuspid valve dysplasia. Rare and heterogeneous congenital diagnoses were grouped under other rare congenital cardiac diseases, whereas cases with multiple congenital abnormalities were classified as complex congenital cardiac disease. These cases were retained as a separate category rather than being assigned to a single primary lesion, because the hemodynamically dominant defect and the lesion responsible for the murmur could not be determined consistently from the harmonized multicenter dataset. Non-structural cardiac conditions included pulmonary hypertension and tricuspid regurgitation in the absence of a defined structural cardiac disease at the time of data entry. Pulmonary hypertension was assigned when it was recorded as the primary echocardiographic diagnosis by the attending clinician, based on Doppler evidence of increased pulmonary arterial pressure and/or supportive echocardiographic findings such as right-sided cardiac remodeling, interventricular septal flattening, or pulmonary arterial enlargement. Tricuspid regurgitation in the absence of a defined structural cardiac disease was assigned when tricuspid regurgitation was recorded as the main echocardiographic abnormality without concurrent predefined structural cardiac disease, congenital cardiac defect, or pulmonary hypertension. This category was therefore descriptive rather than etiologic and may have included dogs with variable TR severity or uncertain underlying cause. No structural cardiac disease was defined as the absence of clinically relevant morphologic or functional cardiac abnormalities on transthoracic echocardiography, including normal cardiac chamber dimensions, normal systolic function, absence of acquired or congenital structural cardiac disease, absence of pathologic shunting, and no Doppler evidence of clinically relevant outflow tract obstruction. Dogs with an audible murmur but no structural cardiac disease were classified as having an innocent or functional murmur. Unclassified cardiac diagnoses comprised cases that could not be consistently assigned to one of the predefined diagnostic categories after harmonization. A predefined drop-down list of common cardiac diagnoses was provided in the electronic data entry form to minimize spelling errors and variation in diagnostic terminology across centers. Additional free-text fields were available to record diagnoses not included in the predefined list, rare cardiac conditions, and multiple concurrent cardiac abnormalities. Free-text entries were systematically reviewed and harmonized into predefined diagnostic categories where possible; cases that could not be assigned consistently were classified as unclassified cardiac diagnoses. Examples included ambiguous or incompletely specified valvular abnormalities, mixed cardiac diagnoses without a clearly assignable primary condition, and rare echocardiographic abnormalities that did not fit one of the predefined diagnostic categories.

### 2.6 Disease staging

Disease staging was evaluated for dogs with MMVD and DCM. Dogs with MMVD were staged according to established clinical criteria (ACVIM consensus) and categorized as stage B1, B2, or C/D (18). Stages C and D were grouped because treatment status, refractoriness to therapy, and prior vs. current congestive heart failure status were not consistently available as standardized variables across participating centers. Therefore, this category was interpreted as advanced clinical disease rather than as a detailed therapeutic subclassification. For the purposes of this study, dogs with echocardiographic evidence of MMVD but no audible murmur and no echocardiographic evidence of cardiac remodeling were retained in the B1 category, reflecting an echocardiography-based operational classification of preclinical MMVD without remodeling.

For the present analyses, dogs with DCM were assigned to an operational, disease-specific staging framework adapted from published DCM screening recommendations rather than to a formally validated staging system. Categories comprised stage B1 (arrhythmias without an echocardiographic DCM phenotype), stage B2 (an echocardiographic DCM phenotype with or without arrhythmias), and stage C/D (previous or current signs of congestive heart failure) (12). Accordingly, stage-based comparisons should be interpreted within the context of this operational classification. Dogs without available disease stage information were excluded from stage-based analyses but retained in overall descriptive analyses.

### 2.7 Data handling and case selection

To ensure consistency and avoid duplication across study phases, a PMI-prioritized per-dog dataset was constructed, with each dog contributing exactly one data entry to the final analysis. For dogs with an audible heart murmur, the PMI was recorded as the representative auscultation site. Dogs without an audible murmur (grade 0) were assigned to a separate no-murmur category contributing a single non-PMI entry to the dataset.

Descriptive statistics were used to summarize PMI distributions and heart murmur grades across cardiac diagnoses. For MMVD and DCM, murmur grading was evaluated in relation to disease stage. Results are presented as absolute numbers and percentages. For the heatmap of PMI localization, percentages were calculated within each diagnostic category among dogs with an audible murmur, so that each row summed to 100%. This row-wise normalization was used to compare the relative distribution of PMI locations between diagnoses despite differences in group size.

Associations between disease stage and murmur grade distribution were assessed using Pearson's chi-square test for MMVD. For DCM, where several contingency table cells contained low expected frequencies, a Monte-Carlo approximation of the Fisher–Freeman–Halton exact test was applied. For selected comparisons, odds ratios (ORs) with 95% confidence intervals (CIs) were calculated. A two-sided *p*-value < 0.05 was considered statistically significant.

Different analyses were performed on predefined subsets of the study population. Unless otherwise stated, descriptive analyses were based on the complete PMI-prioritized per-dog dataset (*n* = 6,996). PMI localization analyses included only dogs with an audible heart murmur, whereas breed analyses were restricted to dogs enrolled during the expansion phase, for which breed information was available.

## 3 Results

### 3.1 Study population

A total of 6,996 dogs from 14 countries were included in the final PMI-prioritized per-dog dataset (Figure 1), with 2,027 enrolled during the initial phase and 4,969 during the expansion phase. Dogs were enrolled in Germany (*n* = 1,622), Sweden (*n* = 1,147), Poland (*n* = 853), Belgium (*n* = 610), Austria (*n* = 494), Italy (*n* = 436), Spain (*n* = 391), the United States (*n* = 315), Norway (*n* = 290), France (*n* = 230), Denmark (*n* = 224), Bulgaria (*n* = 193), Croatia (*n* = 162), and Greece (*n* = 29). Overall, 5,517 dogs were diagnosed with cardiac abnormalities based on echocardiographic findings, whereas 1,479 dogs had no structural cardiac disease on echocardiography. Among dogs with cardiac abnormalities (*n* = 5,517), MMVD was the most common diagnosis (*n* = 4,527; 82.1%), followed by unclassified cardiac diagnosis (*n* = 319; 5.8%), DCM (*n* = 120; 2.2%), tricuspid regurgitation (*n* = 59; 1.1%), and pulmonary hypertension (*n* = 26; 0.5%).

**Figure 1:**
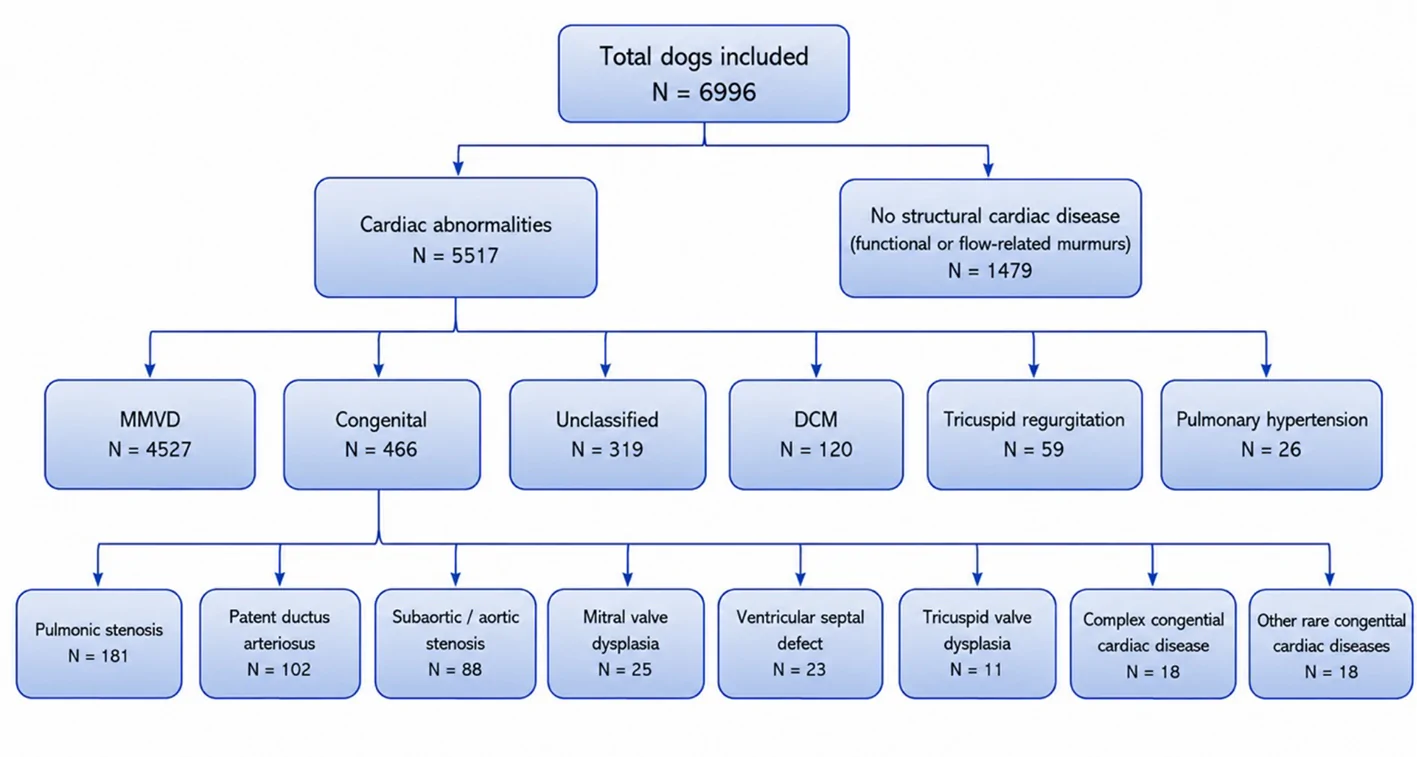
Flowchart of the study population based on a point of maximal intensity (PMI)-prioritized per-dog dataset. A total of 6,996 dogs were included and classified as having either cardiac abnormalities or no structural cardiac disease. Cardiac abnormalities comprised structural cardiac diseases, including myxomatous mitral valve disease (MMVD), dilated cardiomyopathy (DCM), and congenital cardiac disease, such as pulmonic stenosis, patent ductus arteriosus, subaortic/aortic stenosis, ventricular septal defect, mitral valve dysplasia, tricuspid valve dysplasia, complex congenital cardiac disease, and other rare congenital cardiac diseases, as well as non-structural cardiac conditions associated with abnormal echocardiographic findings (pulmonary hypertension and tricuspid regurgitation). No structural cardiac disease was defined by a normal echocardiogram irrespective of murmur presence. Cases that could not be assigned to a predefined diagnostic category were classified as unclassified cardiac diagnoses.

Congenital heart disease was identified in 466 dogs. The most common congenital diagnoses were pulmonic stenosis (PS; *n* = 181; 38.8%), patent ductus arteriosus (PDA; *n* = 102; 21.9%), and subaortic/aortic stenosis (SAS/AS; *n* = 88; 18.9%). Less frequent congenital conditions included mitral valve dysplasia (*n* = 25; 5.4%), tricuspid valve dysplasia (*n* = 11; 2.4%), and ventricular septal defect (VSD; *n* = 23; 4.9%). In addition, 18 dogs were classified as having other rare congenital heart diseases (3.9%), defined as isolated congenital abnormalities not falling into the predefined major categories, and 18 dogs were classified as having complex congenital heart disease (3.9%). Other rare congenital heart diseases (*n* = 18) included double-chambered right ventricle or infundibular stenosis (*n* = 5), atrial septal defect or septum primum defect (*n* = 4), mild left ventricular outflow tract obstruction or increased aortic flow velocity without structural valve abnormality (*n* = 3), tetralogy of Fallot (*n* = 1), truncus arteriosus communis (*n* = 1), and additional single cases including arteriovenous fistula, atrioventricular septal defect, double-outlet right ventricle, and Ebstein anomaly. Complex congenital cardiac disease (*n* = 18) comprised dogs with multiple concurrent congenital abnormalities; the most common components were pulmonic stenosis (*n* = 9), ventricular septal defect (*n* = 7), subaortic/aortic stenosis (*n* = 6), mitral valve dysplasia (*n* = 6), and patent ductus arteriosus (*n* = 5).

### 3.2 PMI localization by cardiac abnormality

PMI localization differed between cardiac diagnoses and showed distinct, disease-specific patterns (Table 1, Figure 2).

**Table 1:** Distribution of point of maximal intensity auscultation site and audibility of heart murmurs by echocardiographic diagnosis.

Diagnosis	N	Left apex	Left base	Right apex	Right base	No murmur
MMVD	4527	4174 (92.2%)	124 (2.7%)	63 (1.4%)	8 (0.2%)	158 (3.5%)
DCM	120	87 (72.5%)	1 (0.8%)	1 (0.8%)	0 (0.0%)	31 (25.8%)
Pulmonic stenosis	181	3 (1.7%)	171 (94.5%)	0 (0.0%)	7 (3.9%)	0 (0.0%)
Patent ductus arteriosus	102	6 (5.9%)	91 (89.2%)	1 (1.0%)	1 (1.0%)	3 (2.9%)
Subaortic/aortic stenosis	88	5 (5.7%)	79 (89.8%)	0 (0.0%)	4 (4.5%)	0 (0.0%)
Innocent murmur	68	20 (29.4%)	48 (70.6%)	0 (0.0%)	0 (0.0%)	0 (0.0%)
Ventricular septal defect	23	0 (0.0%)	1 (4.3%)	6 (26.1%)	15 (65.2%)	1 (4.3%)
Mitral valve dysplasia	25	20 (80.0%)	4 (16.0%)	0 (0.0%)	1 (4.0%)	0 (0.0%)
Tricuspid valve dysplasia	11	2 (18.2%)	0 (0.0%)	6 (54.5%)	2 (18.2%)	1 (9.1%)
Complex congenital	18	3 (16.7%)	11 (61.1%)	1 (5.6%)	2 (11.1%)	1 (5.6%)
Other rare congenital	18	3 (16.7%)	11 (61.1%)	3 (16.7%)	0 (0.0%)	1 (5.6%)
Pulmonary hypertension	26	1 (3.8%)	0 (0.0%)	12 (46.2%)	4 (15.4%)	9 (34.6%)
Tricuspid regurgitation	59	3 (5.1%)	0 (0.0%)	38 (64.4%)	7 (11.9%)	11 (18.6%)

Each dog was included once using a PMI-based per-dog dataset. Values are presented as absolute numbers (n) and percentages within each disease. Dogs without an audible murmur were assigned to the “no murmur” category and were not included in PMI localization analyses. Rare isolated congenital cardiac anomalies were grouped as other rare congenital heart diseases, whereas dogs with multiple congenital abnormalities were classified as complex congenital heart disease. PMI, point of maximal intensity; MMVD, myxomatous mitral valve disease; DCM, dilated cardiomyopathy; PS = pulmonic stenosis; PDA, patent ductus arteriosus; SAS/AS, subaortic or aortic stenosis; VSD, ventricular septal defect.

**Figure 2:**
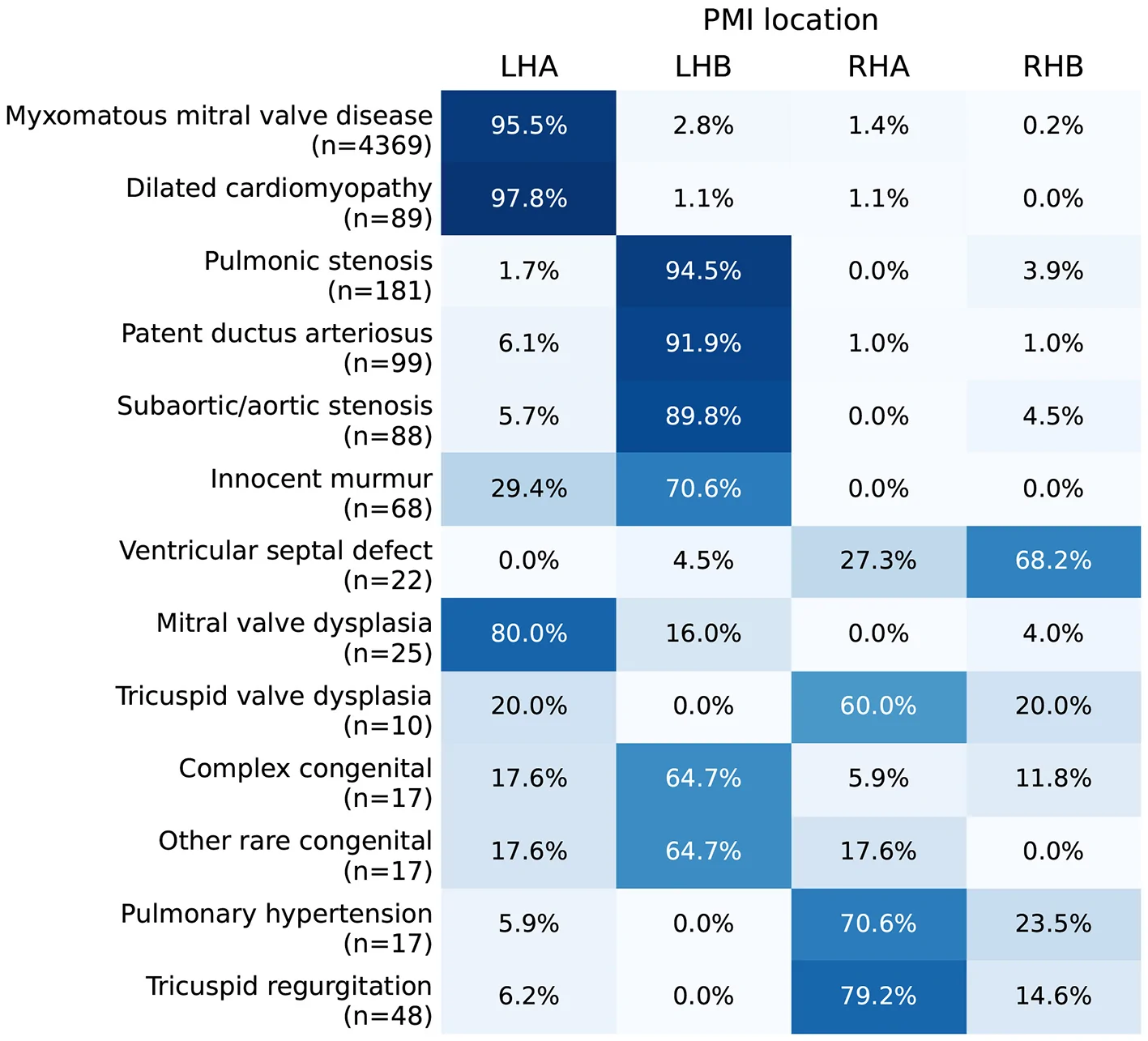
Auscultation site patterns (point of maximal intensity, PMI) by echocardiographic diagnosis. Heatmap illustrating the distribution of the point of maximal intensity (PMI) across four standardized auscultation sites (left heart apex [LHA], left heart base [LHB], right heart apex [RHA], right heart base [RHB]) in dogs with echocardiographically confirmed cardiac diagnoses. Values are expressed as percentages within each diagnostic category (row-wise normalization), with darker shading indicating a higher proportion of dogs with PMI at a given site. Only dogs with an audible heart murmur were included in this analysis. Consequently, the number of dogs differs from the overall disease population reported in Table 1. Numbers in parentheses indicate the number of dogs per diagnosis.

In dogs with MMVD, the PMI was predominantly localized to the LHA, accounting for 95.5% of cases. LHB and right-sided PMI localizations were uncommon. A similar distribution was observed in dogs with DCM, in which the PMI was located at the LHA in 97.6% of dogs with an audible murmur.

Dogs with PS, SAS/AS, and PDA most commonly exhibited a PMI at the LHB, representing 94.5%, 89.8%, and 91.8% of cases, respectively. Apical PMI localization in these diseases was uncommon.

Specific cardiac conditions showed differing patterns of right-sided PMI localization. In dogs with tricuspid regurgitation, the PMI was most frequently localized to the RHA (79.2%), whereas in dogs with VSD, the PMI was predominantly localized to the RHB (68.2%).

### 3.3 Heart murmur grading in relation to disease stage

#### 3.3.1 Myxomatous mitral valve disease

In dogs with MMVD, heart murmur grading was associated with disease stage (Figure 3). In stage B1 (*n* = 2,215), 7.1% of dogs had no audible heart murmur despite echocardiographic evidence of mitral regurgitation. Among dogs with an audible murmur in stage B1, murmurs were most commonly classified as soft (47.8%) or moderate (34.0%), whereas loud murmurs were less frequent (11.2%). In stage B2 (*n* = 1,546), loud murmurs were most common (68.7%), followed by moderate (27.4%) and soft murmurs (3.9%). In stage C/D (*n* = 721), the majority of murmurs were classified as loud (93.9%), with smaller proportions of moderate (5.5%) and soft (0.6%) murmurs. A chi-square test demonstrated a significant association between disease stage and murmur grade distribution (χ^2^ = 2384.4, *df* = 6, *p* < 0.001). When murmur intensity was dichotomized as soft vs. moderate/loud (excluding dogs without an audible murmur), dogs with a murmur greater than soft had higher odds of being in stage B2 or C/D rather than stage B1 (OR 35.88, 95% CI 27.63–46.60; Fisher's exact test, *p* < 0.001).

**Figure 3:**
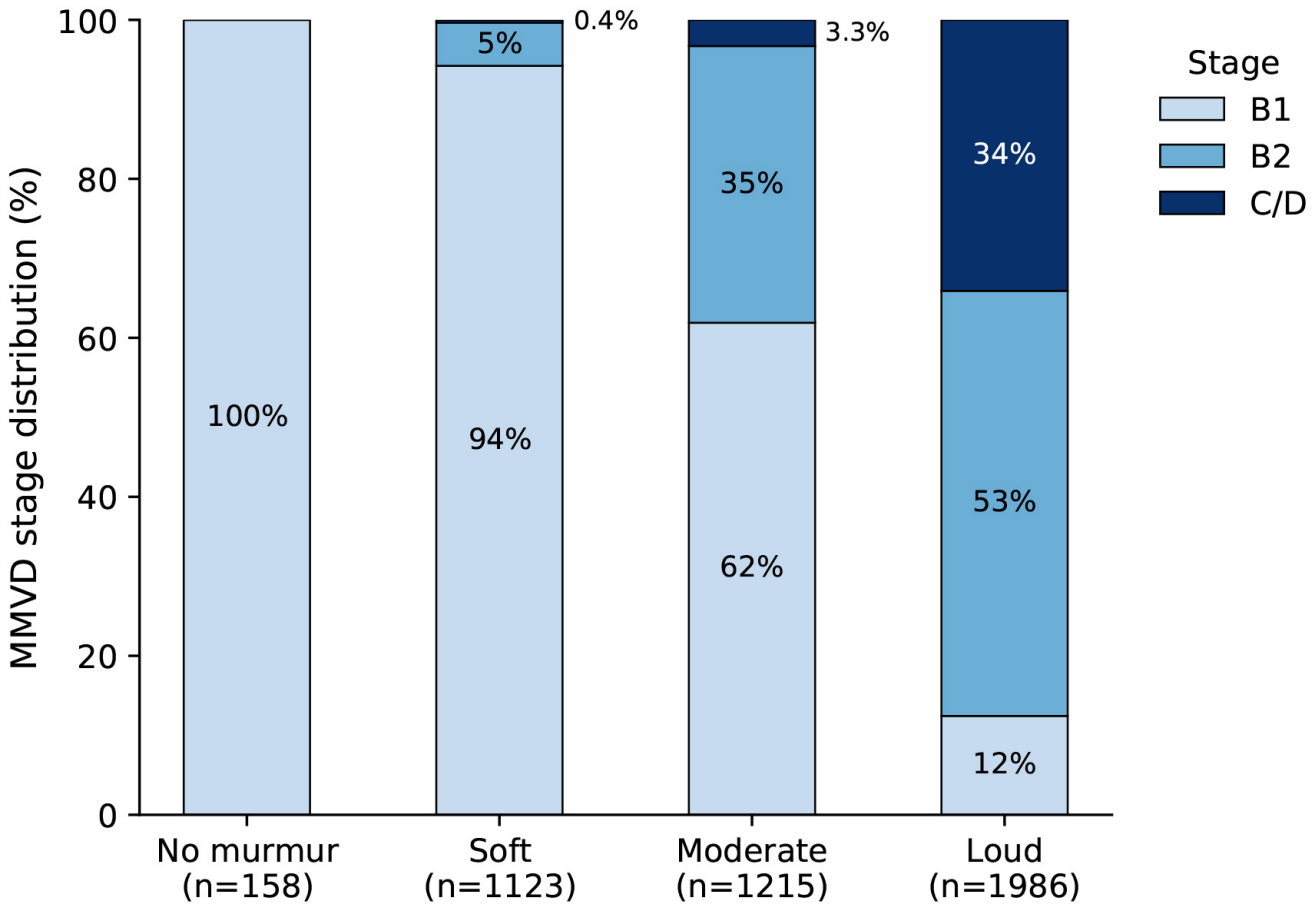
Distribution of MMVD stages according to heart murmur intensity. Bars represent the proportion of dogs in each stage (B1, B2, C/D) within each murmur category (no murmur, soft, moderate, loud). Dogs without an audible murmur but with echocardiographic evidence of MMVD and no cardiac remodeling were included in the B1 category for this analysis. Absolute numbers per murmur category are shown below the x-axis. Only dogs with available stage information were included (*n* = 4,482).

#### 3.3.2 Dilated cardiomyopathy

In dogs assigned to the DCM categories, murmur grade distributions differed across stages but did not show a consistent or monotonic increase with advancing stage (Supplementary Figure S1). In dogs with DCM and an audible murmur, the PMI was predominantly localized to the left heart apex (87/89; 97.8%), whereas left basilar and right apical PMIs were each observed in one dog (1/89; 1.1% each). No dog with DCM had a right basilar PMI. Stage information was available for 102 of 120 dogs (B1, *n* = 10; B2, *n* = 55; C/D, *n* = 37). In stage B1 (*n* = 10), 70% of dogs had no audible murmur, and the remaining dogs had soft murmurs (30%), with no moderate or loud murmurs observed. In stage B2 (*n* = 55), 23.6% of dogs had no audible murmur, and soft murmurs were most common (52.7%), followed by moderate (20.0%) and loud murmurs (3.6%). In stage C/D (*n* = 37), 29.7% of dogs lacked an audible murmur, and murmur grades were more evenly distributed (soft 32.4%, moderate 27.0%, loud 10.8%). A Monte-Carlo approximation of the Fisher–Freeman–Halton exact test indicated a difference in murmur grade distribution across the operational DCM stages (χ^2^ = 13.95, *p* = 0.03). Given the small B1 subgroup (*n* = 10), this result should be interpreted cautiously and does not establish a monotonic relationship between murmur intensity and DCM severity.

### 3.4 Audible vs. non-audible heart murmurs across cardiac abnormalities

The prevalence of audible heart murmurs differed across cardiac diseases (Table 1). In dogs with MMVD, an audible murmur was present in the majority of cases, with only 3.5% of dogs lacking a detectable murmur; all of these dogs were classified as stage B1. In contrast, the absence of an audible murmur was more frequent in DCM, affecting 25.8% of dogs, and was observed across all disease stages. Among dogs with an audible murmur, soft murmurs predominated. Congenital outflow tract diseases, including PS and SAS/AS, were consistently associated with an audible murmur, whereas only a small proportion of dogs with PDA (2.9%) and VSD (4.3%) lacked a murmur. In dogs with pulmonary hypertension or tricuspid regurgitation, absence of an audible murmur was common (34.6% and 18.6%, respectively).

### 3.5 Breed distribution by cardiac abnormalities

Because breed information was collected only during the expansion phase, breed analyses were performed on a subset of the overall study population. Breed distributions across major cardiac diagnoses are summarized in Supplementary Table S1.

In dogs with MMVD, small and toy breeds predominated, with mixed-breed dogs, Chihuahuas, and Cavalier King Charles Spaniels being most commonly represented. In dogs with DCM, large-breed dogs were most frequently observed, particularly Doberman Pinschers. Breed distributions in congenital cardiac diseases were more heterogeneous and varied by diagnosis, with distinct breed associations observed for specific conditions such as SAS/AS, PS, and PDA.

## 4 Discussion

In this large prospective multicenter study, standardized cardiac auscultation demonstrated disease-associated patterns of murmur localization in dogs with echocardiographically confirmed cardiac abnormalities. The PMI differed between cardiac diseases and closely aligned with established auscultation principles. In addition, the association between murmur intensity and disease stage differed fundamentally between MMVD and DCM.

Differences in murmur localization patterns were observed across cardiac abnormalities. For several diseases, murmurs were strongly clustered to a single auscultation site, with more than 80% of cases sharing the same PMI. This was particularly evident for MMVD, where 95.5% of dogs with an audible murmur had a PMI at the LHA, confirming previous descriptions in a substantially larger prospective cohort (6, 7, 9, 20). Similarly, PS, SAS/AS, and PDA were predominantly associated with a LHB PMI, (2, 5, 21) whereas mitral valve dysplasia was most commonly associated with a LHA PMI. These findings are consistent with the anatomical origin of turbulent blood flow and established auscultatory characteristics of these diseases.

Compared with left-sided murmurs, PMI localization for right-sided cardiac abnormalities appeared less homogeneous, consistent with findings reported in previous veterinary cardiology studies (14, 22–24). Nevertheless, distinct patterns were still observed: VSDs were most frequently associated with a RHB PMI, whereas tricuspid regurgitation and tricuspid valve dysplasia were more commonly associated with a RHA PMI. This may reflect less distinct auscultatory landmarks on the right hemithorax, as well as variation in lesion characteristics and flow dynamics. Despite this overlap, the distinction between right apical and right basilar PMI locations remained clinically relevant and may aid in differentiating between right-sided cardiac abnormalities.

Several cardiac abnormalities showed a more heterogeneous distribution of murmur localization or a higher proportion of cases without an audible murmur. Pulmonary hypertension demonstrated considerable variability in PMI location, and more than one third of affected dogs lacked a detectable murmur, findings that are in agreement with previous reports (15, 25). In dogs with pulmonary hypertension, audible murmurs are most plausibly related to secondary tricuspid regurgitation or, less commonly, pulmonary regurgitation; however, the present dataset did not allow consistent assignment of murmur origin in individual cases. Similarly, a substantial proportion of dogs with DCM were murmur-negative. This finding is consistent with the underlying pathophysiology, as dogs with DCM may have impaired left ventricular systolic function without substantial valvular regurgitation, resulting in limited turbulent flow and, consequently, absent or low-intensity murmurs (11, 12).

The observed PMI patterns were generally consistent with classic descriptions of canine cardiac auscultation. However, much of the historical support for these principles has been derived from small case series, teaching texts, or expert opinion (1–15). The contribution of the present study is therefore not the proposal of new auscultatory locations, but the prospective quantification of their frequency and variability across a large international multicenter cohort paired with echocardiographic diagnoses.

### 4.1 Heart murmur grading and disease stage in MMVD

In dogs with MMVD, murmur intensity was associated with disease stage, with murmurs becoming progressively louder from stage B1 to B2 and C/D. Loud murmurs predominated in advanced stages. When murmur intensity was dichotomized as soft vs. moderate/loud, dogs with a murmur greater than soft had markedly increased odds of being in stage B2 or C/D rather than stage B1 (OR 35.9, 95% CI 27.6–46.6; *p* < 0.001). These findings are consistent with previous studies demonstrating an association between murmur intensity and echocardiographic disease severity (6, 7, 9, 20, 26). Increasing murmur intensity has been associated with cardiac remodeling and a higher likelihood of congestive heart failure, whereas soft murmurs are typically indicative of mild, preclinical disease. The progressive increase in murmur intensity with advancing MMVD stage is most likely explained by increasing mitral regurgitant volume and more pronounced turbulent systolic flow across the regurgitant orifice as valvular degeneration and cardiac remodeling progress. However, murmur intensity is not determined by regurgitant volume alone and may also be influenced by jet direction, loading conditions, thoracic conformation, and rhythm-related beat-to-beat variation.

In the present study, 7.1% of dogs with stage B1 MMVD lacked an audible murmur despite echocardiographic confirmation of MR. This finding may reflect the high sensitivity of contemporary echocardiographic equipment, which can detect mild MR that does not generate an audible murmur during routine auscultation. The lack of an audible murmur in dogs with early MMVD has also been reported in previous studies (8, 27, 28). All MMVD dogs without an audible murmur had no echocardiographic evidence of cardiac remodeling and were therefore retained in the B1 category (18).

A substantial proportion of dogs with stage B1 disease presented with murmurs louder than soft. Specifically, 62% of dogs with moderate murmurs and 12% of those with loud murmurs were classified as stage B1. These findings demonstrate that murmur intensity alone cannot reliably determine disease stage and support the use of echocardiographic examination before initiating medical therapy in dogs with suspected MMVD (7, 18).

### 4.2 Heart murmur grading and disease stage in DCM

In contrast to MMVD, dogs with DCM frequently lacked an audible heart murmur across all disease stages. Overall, 25.8% of dogs with echocardiographically confirmed DCM had no detectable murmur, and this was observed in stages B1, B2, and C/D. Among dogs classified as stage C/D, 29.7% lacked an audible murmur on auscultation. These findings are consistent with previous reports describing the frequent absence of murmurs in dogs with DCM (10–13). Although murmur grade distributions differed across the operational DCM stages (Monte Carlo approximation of the Fisher–Freeman–Halton test, *p* = 0.03), murmur intensity did not demonstrate a consistent or monotonic stage-dependent increase comparable to that observed in MMVD; this finding should be interpreted cautiously because the B1 subgroup comprised only 10 dogs. When present, murmurs were most often soft, consistent with the pathophysiology of DCM, in which systolic dysfunction and secondary atrioventricular valve regurgitation commonly limit the generation of high-velocity turbulent flow, resulting in absent or low-intensity murmurs [(12), 36]. However, loud murmurs were identified in six dogs (5.8% overall), representing an atypical finding in DCM. Although this may reflect heterogeneity in disease etiology and phenotypic expression, resulting in variable degrees of secondary valvular regurgitation, the absence of etiologic data precludes any definitive explanation.

Overall, murmur grading showed limited stage-related information in this DCM cohort and should not be used as a surrogate measure of DCM severity or progression. Clinically, the absence of an audible murmur in dogs with DCM should not be considered reassuring. These findings underscore the importance of echocardiographic evaluation for accurate diagnosis and staging and support the use of adjunctive diagnostic tools, such as cardiac biomarker testing or thoracic radiography, when echocardiography is not immediately available (13).

### 4.3 Murmur audibility across cardiac diseases

Pronounced differences in murmur audibility were observed across cardiac diseases and conditions. An audible murmur was present in most dogs with MMVD, whereas absence of a murmur was common in DCM and occurred across all disease stages. Congenital outflow tract lesions and VSDs were almost uniformly associated with an audible murmur, reflecting fixed turbulent blood flow (1–5, 14). Because standardized lesion-level hemodynamic variables were not available for these cases, the reason for the absence of an audible murmur cannot be determined retrospectively. Potential explanations include small or hemodynamically atypical defects, examiner-related detection variability, or limitations of the harmonized dataset. Tricuspid regurgitation showed an intermediate pattern, as murmurs were absent in a subset of dogs, and the PMI, when present, was most often localized to the right heart apex. This finding is consistent with the expected auscultatory localization of right-sided atrioventricular valve regurgitation. However, the tricuspid regurgitation category in the present study was descriptive rather than etiologic and should therefore be interpreted cautiously. It included dogs in which TR was recorded as the main echocardiographic abnormality in the absence of another predefined cardiac diagnosis, but standardized TR jet size, severity, and mechanism were not consistently available. Consequently, TR severity, underlying mechanisms, and clinical relevance could not be assessed systematically. Overall, murmur audibility differed substantially among diagnostic categories. Audible murmurs were common in MMVD and congenital outflow tract disease but were less consistently recorded in myocardial disease and in conditions in which turbulent flow may be limited or variable. Because diagnostic accuracy measures were not calculated, these descriptive differences should not be interpreted as estimates of sensitivity or screening performance.

## 5 Limitations

This study has some limitations. Although examinations were performed prospectively using a standardized auscultation protocol, data were collected by multiple clinicians across different centers, which may have introduced interobserver variability in murmur detection, PMI localization, and murmur grading. Auscultatory findings were clinician-recorded and were not centrally validated using digital sound recordings or objective acoustic analysis. Investigators were not blinded to signalment or clinical context at the time of examination; therefore, expectation bias regarding likely cardiac disease and expected PMI location cannot be excluded. In addition, PMI was recorded by broad thoracic region rather than by exact intercostal space, which may have reduced anatomical precision, particularly in small dogs. Accordingly, the PMI findings should be interpreted as regional clinician-recorded auscultation patterns rather than objective acoustic localization measurements. No center-level effects were modeled, so systematic differences among clinicians or centers cannot be separated from disease-associated patterns. At the same time, the multicenter design reflects real-world clinical cardiology practice and may enhance external validity. The study was conducted in two consecutive phases using two different stethoscope systems. Although murmur grading was performed without digital amplification, subtle differences in sound acquisition between devices cannot be completely excluded. Investigators were required to use the study-provided stethoscope systems rather than their own devices to standardize auscultation across centers and allow digital sound recording. The digital recordings were not used for murmur grading, PMI determination, or any analyses reported in the present study. Because many participating veterinarians routinely use Littmann cardiology stethoscopes in clinical practice, the requirement to use the study-provided Littmann-based stethoscope systems was unlikely to represent a major change in auscultation technique for most investigators. Nevertheless, use of a study-specific stethoscope system may have influenced individual examiner perception compared with each clinician's own routinely used stethoscope.

A PMI-prioritized per-dog dataset was used, with each dog contributing a single data point to the analysis. While this ensured methodological consistency and avoided overrepresentation of individual dogs, it does not capture the full variability of multisite auscultation. Disease stage information was not available for all dogs, particularly in the DCM cohort, limiting stage-based analyses to subsets of the study population. In addition, auscultation was restricted to four predefined thoracic regions; carotid or cranial auscultation sites were not included, and differentiation between pulmonic and aortic stenosis based on radiation of the murmur was therefore not assessed. Subcostal Doppler views were not mandated as a uniform protocol component; therefore, underestimation or misclassification of mild outflow tract obstruction cannot be completely excluded. For congenital heart diseases, lesion-specific severity variables such as Doppler-derived pressure gradients, shunt ratios, or standardized measures of hemodynamic relevance were not consistently available in the harmonized dataset; therefore, associations between murmur intensity and congenital disease severity could not be evaluated. Detailed breed information was only available for dogs enrolled during the expansion phase, and the indication for echocardiography was not recorded as a standardized variable. Therefore, breed distributions may have been influenced by referral and screening practices and should not be interpreted as breed-specific prevalence estimates. Because the study population consisted primarily of dogs evaluated by cardiology-trained clinicians, the findings may not be directly generalizable to primary care settings. Sedation status and drug protocols for echocardiography were not recorded, so any influence of sedation on echocardiographic measurements or diagnostic classification could not be evaluated; however, auscultation was completed before any sedation. For dogs with MMVD and DCM, stages C and D were analyzed together, and treatment status was not consistently available. Therefore, potential effects of diuretic therapy, previous congestive heart failure, or refractory disease on murmur intensity could not be evaluated separately. Pathological arrhythmias, including atrial fibrillation, were not systematically recorded and therefore could not be evaluated as potential modifiers of murmur intensity, although rhythm disturbances may influence beat-to-beat hemodynamics and auscultatory findings. Finally, the study was descriptive and did not formally evaluate the diagnostic sensitivity, specificity, or predictive values of auscultation relative to echocardiography.

Importantly, this study was not designed as a prevalence study. Dogs were examined in specialized cardiology settings, and inclusion was referral-based. Consequently, the distribution of cardiac abnormalities observed in this cohort should be interpreted as characteristics of the study population rather than population-based prevalence estimates. The relative proportions of congenital and other cardiac diseases may therefore reflect referral bias inherent to specialized centers.

## 6 Conclusions

Taken together, these findings describe the complementary roles of cardiac auscultation and echocardiography in the evaluation of canine cardiac disease. Structured four-point auscultation identified characteristic regional PMI patterns and may inform the initial prioritization of differential diagnoses. Murmur intensity increased across MMVD stages at the group level, while individual overlap remained substantial. In DCM, murmur presence and intensity were inconsistent and often absent, including in advanced disease, and no monotonic stage-related pattern was observed. Auscultatory findings should therefore be interpreted as descriptive clinical information rather than as a substitute for disease classification, severity assessment, or echocardiographic staging.

## Data Availability

The anonymized data supporting the conclusions of this article are available from the corresponding author upon reasonable request. Requests will be evaluated on the basis of the proposed scientific purpose and may be subject to an appropriate data-use agreement.

## Supplementary material

Supplementary Figure S1 Distribution of dilated cardiomyopathy (DCM) stages according to heart murmur intensity. Stacked bars represent the proportion of dogs in stages B1, B2, and C/D within each murmur category (no murmur, soft, moderate, loud). Absolute numbers per murmur category are indicated below the x-axis. Only dogs with available stage information were included (*n* = 102).
