# Supplementary material for: Prospective multicenter study of murmur localization and stage-associated murmur grading in dogs with echocardiographically diagnosed cardiac diseases: the LISTEN study
Source: Front Vet Sci. 2026 Oct 13;13:1944439. doi: 10.3389/fvets.2026.1944439 (PMC13711540; doi:10.3389/fvets.2026.1944439)
Supplement: Supplementary file 1 [file Table_1.docx]

**Supplementary Table S1. Top-five Breed distribution across major canine cardiac diseases including only dogs from the expansion cohort (n=4,969)

Values are presented as absolute numbers (n) and percentages within each cardiac disease.**

| Cardiac disease | Breed | n | % |
| --- | --- | --- | --- |
| MMVD (n = 3069; ¨ | Mixed Breed | 570 | 18.6 |
|  | Chihuahua | 469 | 15.3 |
|  | Cavalier King Charles Spaniel | 285 | 9.3 |
|  | Dachshund | 85 | 2.8 |
|  | Yorkshire Terrier | 74 | 2.4 |
|  | Others | 1586 | 51.7 |
| DCM (n = 103) | Doberman Pinscher | 20 | 19.4 |
|  | Weimaraner | 6 | 5.8 |
|  | Great Dane | 6 | 5.8 |
|  | Mixed Breed | 6 | 5.8 |
|  | Cocker Spaniel | 5 | 4.9 |
|  | Others | 60 | 58.3 |
| Subaortic / aortic stenosis (n = 72) | Golden Retriever | 15 | 20.8 |
|  | Boxer | 7 | 9.7 |
|  | Rottweiler | 3 | 4.2 |
|  | Mixed Breed | 3 | 4.2 |
|  | American Bulldog | 2 | 2.8 |
|  | Others | 42 | 58.3 |
| Pulmonic stenosis (n = 144) | French Bulldog | 19 | 13.2 |
|  | Mixed Breed | 17 | 11.8 |
|  | Boxer | 7 | 4.9 |
|  | Chihuahua | 6 | 4.2 |
|  | Cavalier King Charles Spaniel | 5 | 3.5 |
|  | Others | 90 | 62.5 |
| Patent ductus arteriosus (n = 69) | Mixed Breed | 9 | 13.0 |
|  | Chihuahua | 6 | 8.7 |
|  | Maltese | 5 | 7.2 |
|  | Welsh Corgi | 3 | 4.3 |
|  | Pomeranian | 3 | 4.3 |
|  | Others | 43 | 62.3 |
